# Common bacterial causes of external ocular infections, associated risk factors and antibiotic resistance among patients at ophthalmology unit of Felege Hiwot Referral Hospital, Northwest Ethiopia: a cross-sectional study

**DOI:** 10.1186/s12348-021-00238-2

**Published:** 2021-03-01

**Authors:** Zimam Ayehubizu, Wondemagegn Mulu, Fantahun Biadglegne

**Affiliations:** Department of Medical Laboratory Science, College of Medicine and Health Sciences, Bahir Dar University, Bahir Dar, Ethiopia

**Keywords:** Bacteria, External ocular infections, Antibiotics, Resistance, Multi-drug resistance, Ethiopia

## Abstract

**Background:**

Ocular infections are a serious public health problem in Ethiopia with increased incidence of morbidity and blindness. Empirical therapy with topical ophthalmic broad spectrum antibiotic formulations is also a prevailing practice. The aim of this study was to determine the bacterial causes of external ocular infections (EOIs), antimicrobial resistance and its associated risk factors among patients at Felege Hiwot Referral Hospital, Northwest Ethiopia.

**Methods:**

A hospital based cross - sectional study was conducted from 1 February to 30 April 2019. Patients with EOIs were consecutively included from 1 February to 30 April, 2019. Data were collected using structured questionnaire by face-to-face interview and complemented with patient card review. Conjunctival, eyelid margin and lacrimal sac swabs were collected aseptically. Bacterial species were identified using standard bacteriological techniques. Antimicrobial susceptibility testing was done using Kirby-Bauer disk diffusion method. Binary logistic regression analysis was calculated to identify the factors associated with EOIs. P.value (p) < < 0.05 was considered as statistically significant.

**Results:**

A total of 360 patients took part in the study and majority of them were males (64.7%). The median age of study participants was 59.5 years. Overall, 208(57.8%) (95%CI = 52.6– - 62.8%) of patients had culture confirmed bacterial EOIs. The proportion of culture confirmed EOIs was 60.4% among conjunctivitis cases and 55.8% among blepharitis. Ocular trauma (P < < 0.001), ocular disease (P < < 0.001) and having eye allergy (P = 0.027) were significantly associated with EOIs. The most frequent isolates were S. aureus (37%), K. pneumoniae (13.5%), Proteus(7.2%) spp., S. pneumoniae (4.3%), Citrobacter spp. (3.4%) and P. aeruginosa (2.9%). Gram positive isolates were resistant to penicillin in 87% and ampicillin in 86.2%. Gram negative isolates were resistant to ampicillin (87.5%). P.aeruginosa isolates (85.3%) were resistant to piperacillin and 50% were resistant to tobramycin. Overall, 45.2% of the isolates were multi-drug resistant. The percentage of multi-drug resistance was 80% among Enterobacter isolates and 64.3% among K. pneumoniae.

**Conclusions:**

Bacterial external ocular infections linked with multi-drug resistance and resistance to penicillin, ampicillin, tetracycline and piperacillin are high. Therefore, empirical treatment of eye infections in the study area needs to be monitored by regular antimicrobial-susceptibility testing of isolates.

## Background

Globally, damage of the eye due to ocular infections is responsible for increased incidence of morbidity. Infection and inflammation of the ocular regions may also lead to blindness if prompt and appropriate therapy is not administered [1]. According to World Health Organization estimate, 285 million individuals are visually impaired globally. Among them, 90% are from low-income countries [2]. In the case of sub-Saharan Africa, an estimated 26 million individuals live with visual impairment. Of which, 5.9 million individuals are classified blind [3].

External ocular infections (EOIs) frequently involve the eye lid, conjunctiva and cornea. Bacterial agents are known to cause different types of EOIs. The most common EOIs include conjunctivitis, blerpharitis, keratitis, dacryocystitis and canaliculitis [4, 5]. Such infections are associated with increased incidence of morbidity and blindness worldwide [6, 7].

Gram positive and Gram negative bacteria are major causative agents of ocular infections. The most frequent bacteria associated with ocular infections are *Staphylococcus aureus (S. aureus)*, *Streptococcus pneumoniae (S. pneumoniae), Bacillus, Pseudomonas aeruginosa (P. aeruginosa), Enterobacteriaceae, Neisseria gonorrhoeae, Morraxella* spp*., and Haemophilus influenzae* [8].

According to previous studies in other parts of Ethiopia, 48.8–74.4% of patients had culture confirmed bacterial EOIs [9, 10]. If ocular infections are left untreated, it can damage the structures of the eye leading to visual impairments and blindness. In Ethiopia, 1.6% prevalence of blindness was reported and it was estimated that 87.4% of the cases were due to lack of prompt treatment of microbial infections [9, 10].

Virulence of the pathogen, poor personal hygiene, poor living conditions, decreased immune status, trauma, surgery, chronic nasolacrimal duct obstruction and systemic diseases are major determinants for the occurrence of EOIs [11–13]. Moreover, uses of self-administered eye drops for ophthalmic conditions are common practices in rural communities and can delay institutional effective therapy and negatively impact visual outcomes [13].

The emergence of resistant bacterial strains against the commonly prescribed antibiotics in the hospitals is a worldwide problem [9, 10]. Treatments for most ocular bacterial infections are primarily empiric with broad-spectrum antibiotics. However, widespread and misuse of these antibiotics for bacterial and viral infections or prophylactics has resulted in emerging global increase of antibiotic resistance [14].

Empirical treatments and self-medications with broad-spectrum antibiotics which are responsible for selection of antibiotic resistance are routinely practiced for bacterial ocular infections in Ethiopia [11, 15]. Thus, diagnosis of EOIs is without laboratory confirmation and little is known about specific etiologies in Ethiopia. As a result, patients are mostly managed empirically and multi-drug resistant bacteria isolates such as methicillin-resistant *S*.*aureus* are becoming very serious problem. Moreover, bacterial etiologic agents of ocular infections and its susceptibility profile to various antibiotics vary according to geography, regional location and from hospital to hospital. Therefore, this study identified the bacterial pathogens, determined their resistance profile to the commonly used antibiotics and identified factors associated with EOIs among patients in Ophthalmology unit of Felegehiwot Referral Hospital, Bahir Dar Ethiopia.

## Methods

### Study design, period and area

A cross-sectional study was conducted from February to April, 2019 at Felege Hiwot Referral Hospital, Ethiopia. Felege Hiwot Referral Hospital is one of the biggest tertiary level referral Hospitals in the Region visited by around 7 million peoples. The hospital currently delivers health care services in its different wards, intensive care units and Ophthalmology units with a total of 400 beds and 561 staffs [16]. On average, 100 patients attend daily at the ophthalmology unit for different ophthalmic cases to get secondary eye care, refraction examination, minor and major ocular surgery and other common examinations and treatment services. All patients with external ocular infections at Felege Hiwot Referral Hospital Ophthalmology unit during the study period were the study populations.

### Inclusion and exclusion criteria

Patients who had redness of the eye, discharging, mucoid or mucopurulent secretion and/or conjunctival thickening were included to the study. But patients who received antibiotic treatment for the past 2 weeks and those who had case of keratitis were excluded from the study.

### Variables

Bacteria profile of external ocular infections was the dependent variable while demographic variables (age, sex, and residence, educational and occupational status), types of ocular infections, previous ocular infection, eye surgery, ocular trauma, previous eye allergy and history of self-medication were independent variables.

### Sample size determination and sampling

The sample size was determined using single population proportion formula n = (z)^2^ P (1-p)/ d^2^ where, n = sample size, z = level of confidence according to the standard normal distribution, p = sample proportion and d = tolerated margin of error. Therefore, by taking z = 1.96 for a level of confidence of 95%, *p* = 0.624 which is proportion of external ocular infection taken from previous study in Gondar, Ethiopia [15] and 5% margin of error. Thus, the sample size was calculated as n = (1.96)^2^ × 0.624 (1–0.624)/ (0.05)^2^ = 360. A total of 360 patients included in the study. Convenient sampling technique was used to include the study participants from the study population attending at Ophthalmology unit.

### Data collection

#### Demographic and clinical data

Data on demographic characteristics and history of ocular infection, previous ocular trauma, previous eye surgery, and history of self-medication were collected from each participant by a trained ophthalmic nurse with face-to-face interview and patient card review using a pretested structured questionnaire.

#### Ocular sample collection and transportation

The presence of external ocular infections were clinically assessed by ophthalmologist with thoroughly examination of all patients using a slit lamp microscope [8]. Conjunctival and eyelid swabs were collected using sterile cotton swab pre-moistened with sterile physiological saline [17]. Pus from lacrimal sac was collected using dry sterile cotton swab by applying pressure over the lacrimal sac and allowing the purulent material to reflux through the lacrimal punctum. In cases of acute lacrimal abscess or chronic dacryocystitis, pus was drain and taken on a dry sterile cotton swab [8]. All swabs were transferred into a tube that had 2 ml brain heart infusion broth (Oxoid, UK). All samples were labeled and transported to Microbiology Laboratory of Felege Hiwot Referral Hospital immediately. All ocular samples were collected by the ophthalmologist and ophthalmic nurse.

#### Culture and identification of bacterial isolates

The collected eye swab samples were inoculated from brain heart infusion broth to Blood Agar, Chocolate Agar and MacConkey Agar plates (Oxoid, UK) using sterile wire loops. All the agar plates were then incubated at 37 °C for 24 h. Chocolate agar and Blood Agar plates were incubated within a candle-jar to facilitate CO2 tension. After 24 h of incubation, all plates were examined for bacterial pathogen growth. Identification of bacterial pathogens was made by colony morphology, Gram staining, enzymatic and biochemical tests. Catalase, coagulase, optochin (5 μg) and bacitracin (0.04 μg) tests were applied to identify and differentiate gram positive cocci, while biochemical tests, such as triple sugar iron agar, citrate utilization, oxidase test, lysine decarboxylase agar, urease and indole tests were used to identify gram negative bacterial pathogens [18].

#### Antimicrobial susceptibility testing

Antimicrobial susceptibility testing for all the isolated bacterial species were done on Mueller Hinton agar (Oxoid, UK) by Kirby-Bauer disk diffusion method as per Clinical and Laboratory Standard Institute guideline [19]. The antimicrobial agents tested were penicillin (10 μg), erythromycin (15 μg), clindamycin (2 μg), cefoxitin (30 μg), chloramphenicol (30 μg), amoxicillin-clavulanic acid (30 μg), tobramycin (10 μg), ampicillin (10 μg), gentamycin (10 μg) ciprofloxacin (5 μg), tetracycline (30 μg), trimethoprim sulphamethoxazole (1.25/23.75 μg), piperacillin (100 μg), ceftazidime (30/20 μg), tobramycin (10 μg) (Oxoid, UK). A 0.5 McFarland standard was used to standardized the turbidity of the inoculums suspension. Within 15 min after adjusting the turbidity of the inoculums suspension, a sterile cotton swab was dipped into the adjusted suspension. The dried surface plates were inoculated by streaking the swab over the entire sterile agar surface. The antimicrobial disks were placed on the lawn of bacterial isolates visiting sterile forceps. Inoculated media were incubated at 37 °C for 18–24 h. The diameter of zone of inhibition was determined by using caliper. The results were interpreted using the standard zone sizes of the 2018 Clinical and Laboratory Standard Institute guidelines [19]. All *S*.*aureus* isolates were subjected to cefoxitin disk diffusion test on Mueller Hinton agar plates. Antibiotics were selected based on the isolate type, group of locally available antimicrobials, and local prescribing pattern in Ophthalmology unit. Bacterial isolates that are resistant to three or more antibiotic classes were considered as multi-drug resistant [19].

#### Quality control

Structured questionnaires were used to collect the data. Supervision of the data collection was made regularly on daily basis and the collected data checked for completeness and accuracy. All ocular specimens were collected following standard operating procedures. The sterility of culture media was checked by incubating the media overnight before its use. The performances of all the prepared culture media and biochemical tests were checked by using American Type Culture Collection (ATCC) standard reference strains (*E. coli* ATCC25922, *P. aeruginosa* ATCC27853 and *S. aureus ATCC* 25923). The strains were used as a quality control throughout the study for culture, Gram staining and antimicrobial susceptibly testing.

#### Data analysis

Data were entered and analyzed using Statistical Package for Social Science 23 (IBM Corp Released 2011.IBM SPSS statistics. Armonk, NY: IBM Corp). Descriptive statistics were calculated to describe demographic, bacterial external ocular infections and their antibiotic resistance profiles. Bivariate analysis was done to see the association between dependent and independent variables. To determine independent predictors of bacterial external ocular infections, multivariable logistic regression analysis was done by taking variables whose *p*-value was < 0.21 in the binary logistic regression model. Crude Odds Ratio (COR) and Adjusted Odds Ratio (AOR) with 95% Confidence intervals (CI) were calculated. *P*-value of < 0.05 was considered statistically significant.

#### Ethical consideration

This proposal was reviewed and approved by College of Medicine and Health Science Institutional Review Board. Permission to conduct the study was obtained from FHRH. Detail information including the objective of the study was given to each study participants. Written informed consent was obtained from each study participants and for children assent was obtained from parents before they are asked to give data and sample. Confidentiality of the information kept. Positive findings were reported to ophthalmologists for treatment and any other care.

## Results

### Participants’ characteristics

A total of 360 patients with external ocular infections were enrolled in the study. Of them, 233 (64.7%) were males and 275 (76.4%) were from rural settings. The age range was 4–70 years (Median: 59.5). Two hundred sixty-four (73.3%) of the study groups were illiterate (Table 1).

**Table 1:** Demographic characteristic of patients with EOIs at Felege Hiwot Referral Hospital, 2019

Variables	Number	Percent
Age (in years)		
≤ 5	3	0.83
5–14	4	1.1
15–24	2	0.6
25–34	7	1.9
35–44	35	9.7
45–54	83	23.1
55–64	118	32.8
≥ 65	108	30.0
Sex		
Male	233	64.7
Female	127	35.3
Residence		
Rural	275	76.4
Urban	85	23.6
Religion		
Orthodox	326	90.6
Muslim	34	9.4
Occupation		
Farmer	270	75
Civil servant	11	3.1
Housewife	53	14.7
No job	6	1.7
Business man	15	4.4
Pre-school	5	1.4
Education		
Not read and write	264	73.3
Read and write only	77	21.4
Elementary	5	1.4
High school	4	1.1
College and above	10	2.8
Total	**360**	**100**

### External ocular infections

From a total of 360 patients with external ocular infections, 208 (57.8%) had pathogenic bacteria. The frequency of culture confirmed conjunctivitis, blepharitis and dacryocystitis was 125 (60.4%), 67 (55.8%) and 5 (45.5%), respectively. However, the difference was not statistical significant (*p* = 0.578) (Table 2).

**Table 2:** Culture confirmed external ocular infections and frequency of bacterial isolates among patients at Felege Hiwot Referral Hospital, 2019

Bacterial species	Conjunctivitis (*n* = 207): N (%)	Blepharitis (*n* = 120): N (%)	Blepharo-conjunctivitis: (*n* = 22), N (%)	Dacryocystitis (*n* = 11): N (%)	Total: N (%)
*S. aureus*	55 (26.6)	18 (15)	3 (13.6)	1 (9.1)	77 (37)
CoNS	32 (15.5)	12 (10)	2 (9.1)	2 (18.2)	48 (23.1)
*K. pneumoniae*	12 (5.8)	13 (10.8)	3 (13.6)	0	28 (13.5)
*Proteus* spp.,	5 (2.4)	10 (8.3)	0	0	15 (7.2)
*S. pneumoniae*	5 (2.4)	4 (3.3)	0	0	9 (4.3)
*Citrobacter* spp.,	4 (2.9)	4 (3.3)	0	1 (9.1)	9 (4.3)
*P. aeruginosa*	4(1.9)	1 (0.8)	0	1 (9.1)	6 (2.9)
*Enterobacter* spp.,	3 (1.4)	1 (9.2)	1 (4.5)	0	5 (2.4)
*E. coil*	3 (1.4)	2 (1.7)	0	0	5 (2.4)
*S. pyogenes*	2 (1)	0	2 (9.1)		4 (1.9)
*K. rinoscleromatis*	0	2 (1.7)	0	0	2 (1)
Total Culture positives: N (%)	**125 (60.4)**	**67 (55.8)**	**11 (50)**	**5 (45.5)**	**208 (57.8)**

Key: *CoNS* Coagulase negative Staphylococcus

### Frequency of bacterial isolates

Out of 208 bacterial isolates, 138 (66.3%) were Gram positives. *S. aureus* 77 (37%) was the most frequent isolates with Coagulase negative Staphylococcus 48 (23.1%). The other common isolates were *K. pneumoniae* (13.5%), *Proteus* (7.2%) spp., *S. pneumoniae* (4.3%), *Citrobacter* (4.3%) spp., and *P. aeruginosa* (2.9%). From cases of conjunctivitis and blepharitis, *S. aureus* was the predominant isolate and accounted 26.6% and 15%, respectively. From cases of dacryocystitis, Coagulase negative Staphylococcus (18.2%) was the commonest organism followed by *S. aureus* (9.1%), *Citrobacter* spp., (9.1%) and *P. aeruginosa* (9.1%) (Table 2).

### Multivariable analysis of external ocular infections

On multivariable analysis, external ocular infections was significantly associated with previous ocular disease (AOR = 3.53, 95% CI = 2.175–5.731), eye allergy (AOR = 4.71, 95% CI = 1.191–18.59) and trauma (AOR = 9.97, 95% CI = 4.543–21.9). Patients who had history of ocular disease were 3.5 times more likely to have bacterial external ocular infections compared to the counters. Likewise, patients who had previous eye allergy were 4.7 times more likely to have bacterial external ocular infections compared to those who had no eye allergy. Patients who had trauma were also 10 times more likely to become culture positive for external ocular infections compared to their counter parts (Table 3).

**Table 3:** Bivariable and multivariable analysis of factors associated with bacterial external ocular infection at Felege Hiwot Referral Hospital, 2019

Variables	External Ocular Infection		COR (95% CI)	*P*-value	AOR (95% CI)	*P* value
	Infected	Non-infected				
	N (%)	N (%)				
Sex						
Male	133 (57.1)	100 (42.1)				
Female	75 (59.1)	52 (40.9)	0.92 (0.59–1.43)	0.72	NA	NA
Residence						
Rural	167(60.7)	108 (39.3)	1.66 (1.017–2.707)	0.043	0.68 (0.392–1.190)	0.178
Urban	41 (50.6)	44 (49.4)				
Hospitalization						
Yes	5 (55.5)	4 (44.5)				
No	203 (57.8)	148 (42.2)	1.1 (0.290–4.156)	0.891	NA	NA
Previous ocular disease						
Yes	135 (74.2)	47 (25.8)	4.13 (2.64–6.46)	< 0.001	3.53 (2.175–5.731)	< 0.001
No	73 (41)	105 (59)				
Previous eye surgery						
Yes	39 (81.3)	9 (18.53)	0.27 (0.128–0.582)	0.001	1.63 (0.680–3.921)	0.272
No	169 (54.2)	143 (45.8)				
Previous ocular trauma						
Yes	84 (91.3)	8 (8.7)	12.19 (5.68–26.176)	< 0.01	9.97 (4.543–21.900)	< 0.001
No	124 (46.3)	144 (53.7)				
Presence of eye allergy						
Yes	205 (59)	142 (41)				
No	3 (23)	10 (77)	4.81 (1.3–17.79)	0.019	4.705 (1.191–18.585)	0.027
History of self-medication						
Yes	5 (33.3)	10 (66.7)				
No	203 (58.8)	142 (41.2)	2.86 (0.96–8.54)	0.06	0.61 (0.16–2.29)	0.46

Key: *COR* Crude Odds Ratio, *AOR* Adjusted Odds Ratio, *CI* Confidence interval, *NA* Not applicable

### Antimicrobial resistance profiles of gram positive isolates

Gram positive isolates were resistant to penicillin 120 (87%), ampicillin 119 (86.2%) and tetracycline 72 (52.2%). *S. aureus* isolates revealed 96.1% of resistance to both ampicillin and penicillin. The proportion of methicillin-resistant *S*.*aureus* among the total *S. aureus* isolates was 8 (16.9%). *S. pneumoniae* isolates revealed high (66.7%) percentage of resistance to sulphamethoxazole- trimethoprim. Gram positive isolate revealed low percentage of resistance to ciprofloxacin (9.6%), clindamycin (7.5%) and gentamycin (10.4) (Table 4).

**Table 4:** Antimicrobial resistance profiles of Gram positive isolates from external ocular infections at Felege Hiwot Referral Hospital, 2019

Antimicrobials	*S*. *aureus* (*n* = 77)	CoNS (*n* = 48)	*S*.*pneumoniae* (*n* = 9)	S. pyogenes (*n* = 4)	Total (*n* = 138)
	R%	R%	R%	R%	R%
Ampicillin	74 (96.1)	45 (93.7)	0	0	119 (86.2)
Cefoxitin	8 (16.9)	16 (23.3)	NA	1 (25)	25 (19.4)
Penicillin	74 (96.1)	46 (94.8)	0	0	120 (87)
Sulphamethoxazole-trimethoprim	16 (20.8)	10 (20.9)	6 (66.7)	1 (25)	33 (23.9)
Chloramphenicol	20 (26)	16 (33.3)	3 (33.3)	0	39 (28.3)
Erythromycin	18 (23.4))	8 (12.5)	0	0	26 (18.8)
Gentamycin	7 (9.1)	6 (12.5)	NA	NA	13(10.4)
Tetracycline	35 (45.5)	32 (66.6)	3 (33.3)	2 (50)	72 (52.2)
Ciprofloxacin	3 (3.9)	9 (18.7)	NA	NA	12 (9.6)
Clindamycin	5 (6.2)	3 (6.3)	2 (22.2)	NA	10 (7.5)
Total	**260 /770 (33.8)**	**191 /480 (39.8)**	**14/63 (22.2)**	**4/28 (14.3)**	**469/1380 (34)**

key: *R%* Percent of isolates resistant to the tested drugs, *CoNS* Coagulase negative Staphylococcus

### Antimicrobial resistance profiles of gram negative bacteria isolates

Majority of Gram negative bacterial isolates revealed resistance to ampicillin 56 (87.5%) and tetracycline 36 (56.3%). *K. pneumoniae* isolates revealed 28 (100%) and 17 (60.7%) of resistance to ampicillin and tetracycline, respectively. *Proteus* spp., also revealed 14 (93.3%) of resistance to ampicillin. On the other hand, isolates of *P*.*aeruginosa* revealed 5 (83.3%) of resistance to piperacillin. Moreover, all isolates of *E. coli*, *Enterobacter* spp., and *Citrobacter* spp., were resistant to tetracycline and amoxacillin-clavulanic acid (Table 5). However, only 8 (11.4%) and 15 (21.4%) of gram negative isolates were resistant to ciprofloxacin and gentamycin, respectively (Table 5).

**Table 5:** Antimicrobial resistance profiles of Gram negative isolates from external ocular infections at Felege Hiwot Referral Hospital, 2019

Antimicrobials	*K. pneumoniae (n = 28)*	*Proteus* spp*., (n* = 15*)*	*P.aeruginosa* (*n* = 6)	*E. coli (n* = 5*)*	*Enterobacter spp., (n* = 5*)*	*Citrobacter spp* (*n* = 9*)*	*K.rinoscleromatis (n* = 2*)*	Total (*n* = 64)
	R%	R%	R%	R%	R%	R%	R%	R%
Ampicillin	28 (100)	14 (93.3)	NA	0	4 (80)	8 (88.9)	2 (100)	56 (87.5)
Amoxacillin-clavulanic acid	3 (12.1)	7 (46.7)	**NA**	1 (20)	5 (100)	2 (22.2)	0	18 (28.1)
Ciprofloxacin	3 (10.8)	1 (6.7)	2 (33.3)	0	1 (20)	1 (11.2)	0	8 (11.4)
Trimethprim-Sulfamethozazole	13 (46.5)	5 (33.3)	NA	1 (20)	0	0	1 (50)	20 (31.3)
Tetracycline	17 (60.7)	4 (26.6)	NA	5(100)	2 (40)	6 (66.7)	2 (100)	36 (56.3)
Gentamycin	4 (14.3)	4 (14.3)	3 (50)	1(20)	1 (20)	2 (22.2)	0	15 (21.4)
Ceftazidime	NA	NA	2 (33.3)	**NA**	NA	NA	NA	2 (33.3)
Piperacillin	NA	NA	5 (83.3	**NA**	NA	NA	NA	5 (83.3)
Tobramycin	NA	NA	3 (50)	**NA**	NA	NA	NA	3 (50)
Total	**68/150 (40.5)**	**35/90 (38.9)**	**15/30 (50)**	**8/30 (26.7)**	**13/30 (43.3)**	**19/54 (35.2)**	**5/12 (41.7)**	**163/396 (41.2)**

key: *R%* percent of isolates resistant to the tested drugs, *NA* Not applicable

### Multi-drug resistance profiles of the bacterial isolates

From the total isolated bacterial species, 94 (45.2%) were multi-drug resistant. Only 4 (1.9%) of bacterial isolates were susceptible to all antibiotics tested. The multi-drug resistance percentage of *Enterobacter* spp., *K. pneumoniae*, *S. aureus* and *Proteus* spp., were 4 (80%), 18 (64.3%), 35 (45.5%) and 5 (33.3%), respectively (Table 6).

**Table 6:** Antibiogram of bacterial isolates from external ocular infections at Felege Hiwot Referral Hospital, 2019

Bacterial species	Ro	R1	R2	R3	R4	R5	R6	MDR ≥ 3
*S.aureus* (*n* = 77)	0	14 (18.2)	28 (36.4)	23 (29.9)	10 (12.9)	2 (2.5)	0	35 (45.5)
CoNS (*n* = 48)	2 (4.1)	10 (25)	10 (20.8)	11 (22.9)	5 (10.4)	6 (12.5)	4 (8.3)	26 (54.2)
*S*. *pneumoniae* (*n* = 9)	0	3 (33.3)	5 (55.6)	1 (11.1)	0	0	0	1 (11.1)
*S*. *pyogenes* (*n* = 4)	0	4 (100)	0	0	0	0	0	0
*K*. *pneumoniae* (*n* = 28)	0	7 (25)	3 (10.7)	10 (35.7)	6 (21.4)	2 (7.1)	0	18 (64.3)
*K*. *rinoscleromatis* (*n* = 2)	0	0	0	2 (100)	0	0	0	2 (100)
*Enterobacter* spp. (*n* = 5)	0	0	1 (20)	1 (20)	3 (60)	0	0	4 (80)
*Proteus* spp. (*n* = 15)	1 (6.7)	2 (13.3)	7 (46.6)	4 (26.6)	0	1 (6.7)	0	5 (33.3)
*Citrobacter* spp. (*n* = 9)	1 (11.1)	4 (44.4)	3 (33.3)	1 (11.1)	0	0	0	1 (11.1)
*E*. *coil* (*n* = 5)	0	3 (60)	1 (20)	1 (20)	0	0	0	1 (20)
*P*. *aeruginosa* (*n* = 6)	0	0	5 (83.3)	1 (16.7)	0	0	0	1 (16.7)
Total	**4 (1.9)**	**47 (24)**	**63 (30.3)**	**55 (26.4)**	**24 (11.5)**	**11 (5.3)**	**4 (1.9)**	**94 (45.2)**

CoNS* Coagulase negative Staphylococci, *MDR* Multi-drug resistance, *Ro* susceptible to all antimicrobials tested, *R1, R2, R3, R4, R5, R6* Resistance to one, two, three, four, five and six antibiotics taken from different classes, respectively

## Discussion

Bacterial causes of external ocular infections are a serious health problem and highly associated with resistance to antibiotics in developing countries [11, 15]. It is becoming major public health concern in Ethiopia while 57.8% of patients had conjunctivitis, blepharitis and dacryocystitis in this study. This prevalence is comparable with studies in other parts of Ethiopia (47.7–59.4%) [2, 4, 7, 20] and India (58.8%) [21]. However, higher percentages were found in Egypt (78.7%) [22] and China (82.78%) [1].

Conjunctivitis was the most common types of external ocular infections with blepharitis in the present study. This was consistent with reports from other parts of Ethiopia [7, 10, 23]. Moreover, blepharitis was the most common ocular infection with conjunctivitis in other areas of Ethiopia [3, 15].

Indentifying the etiology and typical infectious organisms of dacryocystitis is important for the optimal management of patients [24]. In this study, 45.5% of clinically diagnosed dacrocyocystitis cases were caused by aerobic bacteria which are parallel with previous studies in Iran and Israel [20, 24]. The remaining culture negative cases of dacryocystitis might be because of anaerobic and fastidious bacteria or fungal etiologic agents [20, 24].

In this study, Gram positive cocci bacteria shared 66.3% of external ocular infections. This is parallel with other studies in Ethiopia (61.5% & 52%) [10, 11], Egypt (58.9%) [22] and China (78.4%) [1]. The predominance of Gram positive cocci might be due to contamination of the injured eye from skin floras. In addition, *S. aureus* was the most frequent etiology of conjunctivitis, blepharitis, and blepharo-conjunctivitis in the present study. This is consistent with similar studies done in Ethiopia [11, 23], Nigeria [25] and United Kingdom [26]. Anatomical disruption during cataract extraction and lens implantation might be a good opportunity for *Staphylococci* to elicit infection.

In this study, 27.3% and 18.2% of dacryocystitis was caused by Gram positive and Gram negative bacteria, respectively. The predominance of Gram positives over Gram negative bacteria could be due to more number of chronic than acute dacryocystitis cases. This finding is consistent with a study done in Iran where 54% of dacryocystitis were due to Gram positive bacteria while 24% were due to Gram negative bacteria [24]. However, in a similar study conducted at Israel, 61% of dacryocystitis was caused by Gram negative bacteria while 39% were due to Gram positive bacteria [20]. This showed that the etiologic agents of dacryocystitis vary from study area to study area and clinical presentations of dacryocystitis.

The proportion of external ocular infections caused by Gram negative bacteria (33.3%) in the present study was consistent with other reports from Ethiopia (38.5%) [7] and India (35%) [27]. Among Gram negative bacteria isolates, *K. pneumoniae* was the leading isolate in the present study. This is concurrent with studies in Egypt [25] and Libya [28]. But in Saudi Arabia [5] and other parts of Ethiopia [11, 15], *P. aeruginosa w*as the most frequent isolate. In contrast, *E. coli* was reported as dominant bacteria from Gondar, Ethiopia [29].

The commonest organism isolated from cases of dacryocystitis in the present study was coagulase negative Staphylococcus that was isolated in 2 (18.2%) of positive cultures. *S. aureus*, *Citrobacter* spp., and *P. aeruginosa* accounted 9.1% each of the positive cultures. This is almost similar with a study done in Iran [24]. However, in a study done at Israel [20], *Klebsiella* was the commonest organism followed by *E. coli*, *P. aeruginosa*, *Citrobacter* and *Enterobacter*.

In this study, gram positive bacterial isolates revealed high percentage of resistance to ampicillin (86.3%) and penicillin (86.9%). This might be due to earlier exposure of the isolates to these drugs. These drugs are very common and patients can easily access them from pharmacies without a prescription. Similarly, 69.9–81.5% and 73.9–100% level of resistance to ampicillin and penicillin, respectively have been reported in other parts of Ethiopia [7, 11, 12].

*S. aureus* isolates were highly resistant to ampicillin (96.1%) and penicillin (96.1%). This finding is concurrent with other study from Ethiopia where 96.9% of *S*.*aureus* isolates were resistant to ampicillin and penicillin [4]. The highest resistance percentage of *S. aureus* to penicillin and ampicillin might be due to production of beta-lactamase enzymes and alteration of the penicillin binding proteins [30].

The proportion of external ocular infections with methicillin-resistant *S*.*aureus* was 16.9% in the present study. This indicates the spreading of superbugs among outpatients in Ethiopia. The result is variable with previous reports from Uganda (31.9%) [31], United Kingdom (8.3%) [26], and United States (3–64%) [32].

In this study, majority (66.6%) of *S. pneumoniae* isolates were resistant to sulphamethoxazole- trimethoprim. This might be due to mutations in the dihydrofolate reductase and dihydropteroate synthetase genes [32]. Similar findings were reported in Hawassa, Ethiopia [16] and Nigeria [25] where 65% and 75% of *S. pneumoniae* isolates were resistant to sulphamethoxazole- trimethoprim, respectively.

All *K. pneumoniae* isolates of the present study were resistant to ampicillin. *Proteus* spp., *Citrobacter* spp., and *Enterobacter* spp., revealed 80–93.3% of resistance to ampicillin. This might be due to that these bacteria possess beta-lactamases that can confer resistance to ampicillin and many strains have acquired an extended-spectrum beta-lactamases [33]. The range of resistance was consistent with studies from other parts of Ethiopia where 80–100% of the above isolates were resistant to ampicillin [4, 10, 23].

All *E. coli* isolates of the present study were resistant to tetracycline. This might be due to multiple tetracycline efflux pumps and genetic exchange of resistance determinants among various clinical, environmental and commensal bacteria [34]. Similar findings were documented in other parts of Ethiopia [4, 35]. Moreover, higher percentage of *K. pneumoniae* (60.7%) and *Citrobacter* spp. (66.7%) showed resistance to tetracycline in the present study.

Overall, the observed resistance of Gram positive and Gram negative species to different antibiotics could be linked with the empirical prescription of broad spectrum antibiotics, lack of regular screening of antimicrobial resistance, self-medications and misuse of antibiotics in the study area. These are the major contributors for the emergence and spread of multi-drug resistant isolates.

External ocular infections with multi-drug resistant isolates (45.2%) are a major concern in the present study which needs careful handling of eye wounds, trauma or surgery. The percentage is variable with earlier studies in Tigray (53.9%) and Gondar (66.4 and 87%), Ethiopia [9, 12, 15], Saudi Arabia (39%) [5] and China (12.1%) [1]. The percentage of multi-drug resistance isolates of *S. aureus* in the present study (45.5%*)* is lower than a study reported in Gondar, Ethiopia (64.6%) [23]. High percentage of multi-drug resistance was also documented in *K. pneumoniae* (64.3%) and *Enterobacter* spp. (80%) isolates. This could be associated with biofilm formation, transfer of resistance plasmid genes, efflux and production of extended-spectrum beta-lactamases which allows for resistance to multiple antimicrobials [36].

History of ocular trauma was a predictor variable for external ocular infections in this study. This is because mechanical disruption of the conjunctiva or stroma layers of eye allows the skin flora and other contaminants to breach, colonize and initiate infections. The finding was consistent with other studies in Ethiopia [4, 9], Nepal [37], India [38] and Iran [39]. Moreover, previous ocular disease was another predictor variable for the occurrence of external ocular infections in the present study. This finding was supported by other studies in Ethiopia [9], Nepal [37] and Iran [40].

Presence of eye allergy was strongly associated with bacterial external ocular infections in the present study. In most conjunctivitis infection, allergy is one of the major predisposing factors. Itching symptom of allergy might be a cause for skin contaminants to be introduced to the eye and cause chronic bacterial ocular infection [41].

This study was not without limitations because external ocular infections due to anaerobic bacteria, *Chlamydia trachomatis* and molecular mechanism of antimicrobial resistance were not addressed due to the limitations of laboratory setups. Moreover, cases of keratitis which requires special training and experience for corneal scraping were not included.

## Conclusions

Conjunctivitis, blepharitis and dacryocystitis forms of bacterial external ocular infections linked with multi-drug resistance and high levels of resistance to penicillin, ampicillin, tetracycline and piperacillin are prevalent in the study area. Therefore, empirical treatment of eye infections in the study area needs to be guided by antimicrobial - susceptibility testing. Previous ocular diseases, trauma and co-existence of eye allergy were predictor variables for bacterial external ocular infections. Bacterial isolates were susceptible for ciprofloxacin and gentamycin. Further studies on keratitis, intraocular infections and extents of beta-lactamase producing bacterial causes of external ocular infections using molecular techniques are required.

## Data Availability

The finding of this study is generated from the data collected and analyzed based on the stated methods and materials. All the data are already found in the manuscript and there are no supplementary flies. The original data supporting this finding will be available at any time upon request.

## Abbreviations

ATCC: American Type Culture Collection
AOR: Adjusted Odds ratio
CI: Confidence Interval
CONs: Coagulase negative Staphylococcus
COR: Crude Odds Ratio
EOIs: External Ocular Infections

## References

[1] Wang N, Yang Q, Tan Y, Lin L, Huang Q, Wu K (2015). Bacterial spectrum and antibiotic resistance patterns of ocular infection: differences between external and intraocular diseases. J Ophthalmol.

[2] World Health Organization (2019). Blindness and vision impairment.

[3] Schaftenaar E, van Gorp EC, Meenken C, Osterhaus AD, Remeijer L, Struthers HE (2014). Ocular infections in sub-Saharan Africa in the context of high HIV prevalence. Tropical Med Int Health.

[4] Shiferaw B, Gelaw B, Assefa A, Assefa Y, Addis Z (2015). Bacterial isolates and their antimicrobial susceptibility pattern among patients with external ocular infections at Borumeda hospital, Northeast Ethiopia. BMC Ophthalmol.

[5] Ahmed, Essa (2018). Bacterial spectrum of external ocular infections: prevalence and associated in vitro antimicrobial susceptibility and resistance in a tertiary care hospital. In J Adv Res.

[6] HeMavatHi PS, SHenoy P (2014). Profile of microbial isolates in ophthalmic infections and antibiotic susceptibility of the bacterial isolates: a study in an eye care hospital, Bangalore. J Clin Diagn Res.

[7] Aweke T, Dibaba G, Ashenafi K, Kebede M (2014). Bacterial pathogens of exterior ocular infections and their antibiotic vulnerability pattern in southern Ethiopia. Afr J Immunol Res.

[8] Miller JM, Binnicker MJ, Campbell S, Carroll KC, Chapin KC, Gilligan PH (2018). A guide to utilization of the microbiology laboratory for diagnosis of infectious diseases: update by the Infectious Diseases Society of America and the American Society for Microbiology. Clin Infect Dis.

[9] Teweldemedhin M, Gebreyesus H, Atsbaha AH, Asgedom SW, Saravanan M (2017). Bacterial profile of ocular infections: a systematic review. BMC Ophthalmol.

[10] Amsalu A, Abebe T, Mihre A, Delelegn D, Tadess E (2015). Potential bacterial pathogens of external ocular infections and their antibiotic susceptibility pattern at Hawassa University teaching and referral hospital, southern Ethiopia. Afr J Microbiol Res.

[11] Tesfaye T, Beyene G, Gelaw Y, Bekele S, Saravanan M (2013). Bacterial profile and antimicrobial susceptibility pattern of external ocular infections in Jimma University specialized hospital, Southwest Ethiopia. Am J Infect Dis Microbiol.

[12] Muluye D, Wondimeneh Y, Moges F, Nega T, Ferede G (2014). Types and drug susceptibility patterns of bacterial isolates from eye discharge samples at Gondar University hospital, Northwest Ethiopia. BMC Res Notes.

[13] Sreekumar H, Manikandan P, Aiswariya P, Narendran V, Gomathi P, Priya R (2016). Prevalence and antimicrobial susceptibility pattern of staphylococci and streptococci causing ocular infections from a tertiary eye care hospital. South India Biomed Res.

[14] Bertino JS (2009). Impact of antibiotic resistance in the management of ocular infections: the role of current and future antibiotics. Clin Ophthalmol.

[15] Aklilu A, Bitew A, Dessie W, Hailu E, Asamene N, Mamuye Y (2018). Prevalence and drug susceptibility pattern of bacterial pathogens from ocular infection in St. Paul’s hospital millennium medical college, Ethiopia. J Bacteriol Mycol.

[16] FHRH (2018). Annual report of Felegehiwot Referral.

[17] Belyhun Y, Moges F, Endris M, Asmare B, Amare B, Bekele D (2018). Ocular bacterial infections and antibiotic resistance patterns in patients attending Gondar teaching hospital, Northwest Ethiopia. BMC Res Notes.

[18] Cheesbourgh M (2006). District laboratory practice in tropical countries. PartII. 2nded.

[19] Clinical Laboratory Standards Institute (2018). Performance Standards forAntimicrobial Susceptibility Testing; Twenty-Fifth Informational.Supplement.CLSIdocumentM100-S25.

[20] Briscoe D, Rubowitz A, Assia EI (2005). Changing bacterial isolates and antibiotic sensitivities of purulent Dacryocystitis. Orbit.

[21] Okesola AO, Salako AO (2010). Microbiological profile of bacterial conjunctivitis in Ibadan, Nigeria. Ann Ibadan Postgrad Med.

[22] Shahaby AF, Alharthi AA, El Tarras AE (2015). Potential bacterial pathogens of red eye infections and their antibiotic susceptibility patterns in Taif, KSA. Int J Curr Microbiol App Sci.

[23] Getahun E, Gelaw B, Assefa A, Assefa Y, Amsalu A (2017). Bacterial pathogens associated with external ocular infections alongside eminent proportion of multidrug resistant isolates at the University of Gondar Hospital, Northwest Ethiopia. BMC Ophthalmol.

[24] Eshraghi B, Abdi P, Akbari M, Fard MA (2014). Microbiologic spectrum of acute and chronic dacryocystitis. Int J Ophthalmol.

[25] Ubani UA (2009). Bacteriology of external ocular infections in aba, south eastern Nigeria. Clin Exp Optom.

[26] Silvester A, Neal T, Czanner G, Briggs M, Harding S, Kaye S (2016). Adult bacterial conjunctivitis: resistance patterns over 12 years in patients attending a large primary eye care Centre in the UK. BMJ Open Ophthalmol.

[27] Bharathi MJ, Ramakrishnan R, Shivakumar C, Meenakshi R, Lionalraj D (2010). Etiology and antibacterial susceptibility pattern of community-acquired bacterial ocular infections in a tertiary eye care hospital in South India. Indian J Ophthalmol.

[28] Musa AA, Nazeerullah R, Sarite SR (2014). Bacterial profile and antimicrobial susceptibility pattern of anterior blepharitis in Misurata region, Libya. DMR.

[29] Anagaw B, Biadglegne F, Belyhun Y, Mulu A (2011). Bacteriology of ocular infections and antibiotic susceptibility pattern in Gondar University hospital, north West Ethiopia. Ethiop Med J.

[30] Deyno S, Fekadu S, Astatkie A (2017). Resistance of Staphylococcus aureus to antimicrobial agents in Ethiopia: a meta-analysis. Antimicrob Resist Infect Control.

[31] Mshangila B, Paddy M, Kajumbula H, Ateenyi-Agaba C, Kahwa B, Seni J (2013). External ocular surface bacterial isolates and their antimicrobial susceptibility patterns among pre-operative cataract patients at Mulago National Hospital in Kampala, Uganda. BMC Ophthalmol.

[32] Shanmuganathan VA, Armstrong M, Buller A, Tullo AB (2005). External ocular infections due to methicillin-resistant Staphylococcus aureus (MRSA). Eye.

[33] Bush K (2018). Past and present perspectives on β-lactamases. Antimicrob Agents Chemother.

[34] El Moujaber G, Osman M, Rafei R, Dabboussi F, Hamze M (2017). Molecular mechanisms and epidemiology of resistance in Streptococcus pneumoniae in the Middle East region. J Med Microbiol.

[35] Assefa Y, Moges F, Endris M, Zereay B, Amare B, Bekele D (2015). Bacteriological profile and drug susceptibility patterns in dacryocystitis patients attending Gondar University teaching hospital, Northwest Ethiopia. BMC Ophthalmol.

[36] Nirbhavane HM, Bagde US (2017). Resistance by Enterobacter spp. towards several antimicrobial drugs and heavy metals: a review. Afr J Biotechnol.

[37] Markley JL, Wencewicz TA (2018). Tetracycline-inactivating enzymes. Front Microbiol.

[38] Munita JM, Arias CA (2016). Mechanisms of antibiotic resistance. Virulence Microbiol Spectr.

[39] Gautam V, Chaudhary A, Singh SK, Rai PG (2018). Profile of corneal ulcer in a month of harvesting season in a tertiary level eye Hospital of Eastern Nepal. Nepal J Ophthalmol.

[40] Chidambaram JD, Venkatesh Prajna N, Srikanthi P, Lanjewar S, Shah M, Elakkiya S (2018). Epidemiology, risk factors, and clinical outcomes in severe microbial keratitis in South India. Ophthalmic Epidemiol.

[41] Eghtedari M, Beigi V, Mostafavi E (2018). Pediatric microbial keratitis: a tertiary care center report. Shiraz E-Medical J.

